# Supplementary material for: RBD-Based ELISA and Luminex Predict Anti-SARS-CoV-2 Surrogate-Neutralizing Activity in Two Longitudinal Cohorts of German and Spanish Health Care Workers
Source: Microbiol Spectr. 2023 Jan 9;11(1):e03165-22. doi: 10.1128/spectrum.03165-22 (PMC9927417; doi:10.1128/spectrum.03165-22)
Supplement: Supplemental file 1 — Tables S1 and S2. Download spectrum.03165-22-s0001.pdf, PDF file, 0.1 MB [file spectrum.03165-22-s0001.pdf]

RBD-based ELISA and Luminex predict anti-SARS-CoV-2 plasma surrogate-neutralizing activity in two longitudinal cohorts of German and Spanish health care workers

Ruth Aguilar[1], Xue Li[2], Claudia S. Crowell[2], Teresa Burrell[2], Marta Vidal[1], Rocio Rubio[1], Alfons Jiménez[1,3], Pablo Hernández-Luis[4,5], Dieter Hofmann[6,7], Hrvoje Mijočević[6], Samuel Jeske[6], Catharina Christa[6], Elvira D'Ippolito[2], Paul Lingor[8], Percy A. Knolle[7,9], Hedwig Roggendorf[9], Alina Priller[9], Sarah Yazici[9], Carlo Carolis[10], Alfredo Mayor[1], Patrik Schreiner[11], Holger Poppert[12], Henriette Beyer[12], Sophia E. Schambeck[2,12], Luis Izquierdo[1,13], Marta Tortajada[14], Ana Angulo[4,5], Erwin Soutschek[11], Pablo Engel[4,5], Alberto Garcia-Basteiro[1,13,15], Dirk H. Busch[2,7], Gemma Moncunill[1,13], Ulrike Protzer[6,7], Carlota Dobaño[1,13,*#], Markus Gerhard[2,7,*#]

Supplementary material

Supp Table 1. Correlations between antibody levels measured by Luminex and RBD-ACE2 neutralization capacity excluding seronegative samples.

	All samples			Barcelona			Munich		
**Antigens**	**IgA**	**IgG**	**IgM**	**IgA**	**IgG**	**IgM**	**IgA**	**IgG**	**IgM**
N-FL	R= 0.17, p=0.14	R = 0.2, p = 0.012	R = - 0.27, p = 0.32	R = 0.07, p = 0.6	R = 0.23, p = 0.012	R = - 0.25, p = 0.36	R = 0.54, p = 0.034	R = 0.11, p = 0.46	NA
N-Cterm	R=0.12, p=0.31	R = 0.28, p = 0.019	R = 0.091, p = 0.69	R = - 0.055, p = 0.72	R = 0.19, p = 0.36	R = 0.22, p = 0.38	R = 0.53, p = 0.0049	R = 0.21, p = 0.16	R = - 0.8, p = 0.33
RBD	R=0.36, p = 1.4e−05	R = 0.6, p < 2.2e−16	R = 0.27, p = 0.0022	R = 0.29, p = 0.0053	R = 0.59, p = 6.7e−12	R = 0.34, p = 0.00049	R = 0.44, p = 0.0022	R = 0.56, p = 3.4e−05	R = 0.057, p = 0.78

S	R = 0.28,<br>p = 0.00026	R = 0.52,<br>p = 2.6e−13	R = 0.17,<br>p = 0.16	R = 0.23,<br>p = 0.011	R = 0.57,<br>p = 5.7e−12	R = - 0.018,<br>p = 0.9	R = 0.35,<br>p = 0.017	R = 0.53,<br>p = 0.00013	R = 0.45,<br>p = 0.048
S2	R = 0.25,<br>p = 0.0014	R = 0.39,<br>p = 3.3e−08	R = 0.34,<br>p = 0.014	R = 0.24,<br>p = 0.01	R = 0.39,<br>p = 1.8e−06	R = 0.46,<br>p = 0.0036	R = 0.18,<br>p = 0.23	R = 0.44,<br>p = 0.0024	R = 0.081,<br>p = 0.78

Supp Table 2. Correlations of IgG-RBD levels measured by Luminex and ELISA between them, and by ELISA with plasma neutralizing activity excluding seronegative samples.

	**All samples**	**Barcelona**	**Munich**
IgG-RBD Luminex vs ELISA	R = 0.58,<br>p < 2.2e−16	R = 0.63,<br>p = 6.2e−07	R = 0.56,<br>p < 2.2e−16
IgG-RBD ELISA vs %nAb	R = 0.62,<br>p < 2.2e−16	R = 0.76,<br>p < 2.2e−16	R = 0.4,<br>p = 0.0093
